# Association between physical restraint requirement and unfavorable neurologic outcomes in subarachnoid hemorrhage

**DOI:** 10.1186/s40560-021-00541-z

**Published:** 2021-03-12

**Authors:** Kyoko Akiyama, Akihiko Inoue, Toru Hifumi, Kentaro Nakamura, Takuya Taira, Shun Nakagawa, Keisuke Jinno, Arisa Manabe, Sayaka Kinugasa, Hikaru Matsumura, Hajime Shishido, Shota Yokoyama, Tomoya Okazaki, Hideyuki Hamaya, Koshiro Takano, Kazutaka Kiridume, Natsuyo Shinohara, Kenya Kawakita, Yasuhiro Kuroda

**Affiliations:** 1 Department of Nursing, Kagawa University Hospital, 1750-1 Ikenobe, Miki-cho, Kita-gun, Kagawa 761-0793 Japan; 2 Department of Emergency and Critical Care Medicine, Hyogo Emergency Medical Center, 1-3-1 Wakinohamakaiganndori, Chuo-ku, Kobe, Hyogo 651-0073 Japan; 3 Department of Emergency and Critical Care Medicine, St. Luke’s International Hospital, 9-1 Akashi-cho, Chuo-ku, Tokyo, 104-8560 Japan; 4 Emergency Medical Center, Kagawa University Hospital, 1750-1 Ikenobe, Miki-cho, Kita-gun, Kagawa 761-0793 Japan

**Keywords:** Physical restraint, Neurological outcome, Subarachnoid hemorrhage, Delirium

## Abstract

**Background:**

Physical restraint has been commonly indicated to patients with brain dysfunction in neurocritical care. The effect of physical restraints on outcomes of critically ill adults remains controversial as no randomized controlled trials have compared its safety and efficacy, and the association between physical restraint requirement and neurological outcome in patients with subarachnoid hemorrhage (SAH) has not been fully examined. The aim of this study was to examine the association between physical restraint requirement and neurological outcomes in patients with SAH.

**Methods:**

A single-center, retrospective study was conducted on patients with acute phase SAH treated for > 72 h in the intensive care unit from 2014 to 2020. Patients were divided into three groups based on the amount of time required for physical restraint during the first 24–72 h after admission: no, intermittent, and continuous use of physical restraint. Unfavorable neurologic outcome, assessed using the modified Rankin scale upon hospital discharge, has been considered as primary end point.

**Results:**

Overall, 101 patients were included in the study, with 52 patients (51.5%) having unfavorable neurological outcomes. Among them, 46 patients (45.5%) did not use physical restraint, and 55 (54.5%) patients used physical restraint during the first 24–72 h after admission: 26 (25.7%) intermittent and 29 (28.7%) continuous. Multivariable logistic regression analysis showed that continuous use of physical restraint during the first 24–72 h after admission was significantly associated with unfavorable neurological outcomes in patients with SAH (odds ratio, 3.54; 95% confidence interval, 1.05–13.06; *p* = 0.042) compared with no physical restraint.

**Conclusions:**

Continuous use of physical restraint during the first 24–72 h after admission was more significantly associated with unfavorable neurological outcomes than no physical restraint among patients with SAH during the acute phase.

**Supplementary Information:**

The online version contains supplementary material available at 10.1186/s40560-021-00541-z.

## Background

Physical restraint defined as “any manual method, physical, or mechanical device, material, or equipment that immobilizes or reduces the ability of a patient’s movement” have been commonly indicated for patients with brain dysfunction in neurocritical care [1–3]. Its effect on outcomes of critically ill adult patients remains controversial as no randomized controlled trials (RCTs) have compared its safety and efficacy [1, 4].

Agitation, consciousness fluctuation, and delirium after a subarachnoid hemorrhage (SAH) have been associated with unfavorable neurological outcome [5–7]. However, the association between physical restraint requirement and neurological outcomes in patients with SAH has not been fully examined.

Therefore, this study aimed to examine the association between physical restraint requirement and neurological outcome in patients with SAH.

## Methods

### Study design and setting

This single-center, retrospective study was conducted at the Kagawa University Hospital, a 613-bed teaching institution with an 8-bed intensive care unit (ICU) managed by a neurointensivist. Medical records were reviewed with the approval of the institutional review board (approval number: 2020-053) and in accordance with the ethical standards established in the 1964 Declaration of Helsinki and its later amendments. The requirement for patient consent was waived due to the retrospective nature of the study.

### Study participants and inclusion criteria

We included patients aged ≥ 18 years who were consecutively admitted to the ICU between July 1, 2014 and July 31, 2020, with a confirmed diagnosis of aneurysmal SAH. Patients who met the following inclusion criteria were included: acute phase of SAH treated for > 72 h in the ICU. Exclusion criteria were patients who did not undergo treatment (coil or clip) for aneurysmal SAH, patients provided with comfort care only, or patients admitted to the ICU for < 72 h. Additionally, those with Hunt and Kosnik (H & K) grade 5, Richmond Agitation–Sedation Scale (RASS) score of − 5, or neuromuscular blockade use during the first 24–72 h after admission were excluded.

### General management of SAH in the ICU

All patients were managed in accordance with Guidelines for the Management of Aneurysmal Subarachnoid Hemorrhage by the American Heart Association/American Stroke Association [8]. In addition to the general intensive care, all patients were monitored for clinical deterioration or cerebral infarction development due to delayed cerebral ischemia (DCI). Fever was treated aggressively with acetaminophen, nonsteroidal anti-inflammatory medications, or cooling devices.

### Analgosedation management

A meeting was held every morning and evening, and the depth of sedation was determined in patients undergoing mechanical ventilation. The depth of sedation was assessed using the RASS [9, 10]. If increased intracranial pressure was noted or highly suspected, deep sedation targeting a RASS score of − 5 with neuromuscular blockade was initiated as needed. The minimum amount of sedatives, such as propofol, midazolam, or dexmedetomidine (DEX), which were necessary to prevent ventilator dyssynchrony and patient discomfort, were used. Analgesics, including acetaminophen, nonsteroidal anti-inflammatory medications, and fentanyl, were administered as required. Each physician was responsible for the choice of sedatives.

### Physical restraint

Physical restraint was initiated based on the RASS score. Examples of physical restraints include Mitten restraints, vests, straps/belts, limb ties, and bedside rails [11].

RASS − 3 to − 1: Delirium is assessed using the Confusion Assessment Method for the ICU (CAM-ICU). Physical restraint is considered in patients receiving mechanical ventilation to prevent accidents.

RASS 0: Basically, physical restraint was initiated, but only occasionally when patients are expected to experience delirium at night time to prevent accidents.

RASS ≥ 1: Analgesia and sedative drugs were titrated to obtain the RASS-2 in patients receiving mechanical ventilation. In patients expected to pull or remove tubes or catheters, Mitten restraints were used in addition to limb ties. Further, application of vests was considered to prevent accidental events.

### Assessment of delirium in SAH patients in the ICU

Delirium was assessed by ICU nurses. ICU patients were routinely assessed for the occurrence of delirium through the CAM-ICU. In addition, delirium was defined based on a positive RASS score, or bedside nurse’s judgments according to the presence of agitation, hallucination, dangerous behavior, and use of pharmacologic treatments for agitation and hyperactive delirium such as antipsychotic drugs (risperidone or haloperidol). During the ICU stay, delirium was assessed three times a day and when patient’s status changed. Delirium during the first 48–72 h after admission was obtained to clarify the phase differences between physical restraint requirement and delirium development.

### Data sampling

The following data were collected: age, sex, H & K grade, Fisher score, World Federation Neurological Surgeons (WFNS) grade, treatment modality (coil or clip), sedatives and analgesia administration, RASS score, physical restraint duration during the first 24–72 h after admission (no, intermittent, and continuous use of physical restraint), delirium during the first 48–72 h after admission, laboratory data on admission, aneurysm location [12], modified Rankin scale (mRS) upon hospital discharge, DCI rate, mechanical ventilation duration, ICU stay duration, hospital stay duration, and hospital mortality.

The physical restraint duration was determined during the first 24–72 h after admission because various treatment strategies (emergency surgical operation or angiography with general anesthesia, deep sedation followed by treatment on the next day, computed tomography followed by treatment, and so on) were initiated for the first 24 h; therefore, this phase was excluded in the present study.

Continuous physical restraint was defined as the patients who required continuous physical restraint during the first 24–72 h after admission. Intermittent physical restraint was defined as the patients who required any physical restraint during the first 24–72 h after admission.

### Outcome measures

Unfavorable neurologic outcome was considered as the primary end-point, which was assessed using the mRS upon hospital discharge [13]. mRS is a global disability measurement tool and comprises seven outcome categories: no symptoms at all, no significant disability, slight disability, moderate disability, moderately severe disability, severe disability, and death. All patients with SAH were examined in real time using mRS upon hospital discharge and listed their medical records. The neurologic outcome was defined as unfavorable when the mRS score was 3–6 and as favorable when the mRS score was 0–2. The secondary outcome was the occurrence of delirium evaluated by ICU nurses.

### Statistical analysis

To obtain the association between physical restraint in the acute phase and neurological outcomes, patients were divided into three groups based on the amount of time required for physical restraint during the first 24–72 h after admission: no, intermittent, and continuous use of physical restraint. Demographic factors and baseline characteristics were summarized using descriptive statistics. The groups were compared using Kruskal–Wallis test or Mann–Whitney U test, and categorical comparisons were drawn using the Fisher’s exact test or chi-square test, as deemed appropriate. Univariate and multivariate analyses were performed to explore independent factors that predicted the unfavorable neurologic outcomes. Covariates of age (> 65 years), H & K grade, treatment modality (coil or clip), aneurysm location (anterior cerebral arteries or not), and physical restraint were included in the multivariable analysis. The Cochran–Armitage trend test was used to examine the trend between physical restraint during the first 24–72 h after admission and incidence of delirium during the first 48–72 h after admission. Subgroup analysis was performed on patients with RASS of ≥ − 2 and Hunt & Kosnik grade of 1–3. We also repeated the analysis using different definitions of physical restraint during the first 24–72 h after admission as a continuous variable. Statistical analyses were performed using the JMP version 12 statistical software (SAS Institute, Cary, NC, USA). A two-sided *p* value < 0.05 was considered statistically significant for all analyses. Missing data were not replaced or estimated.

## Results

**Fig. 1:**
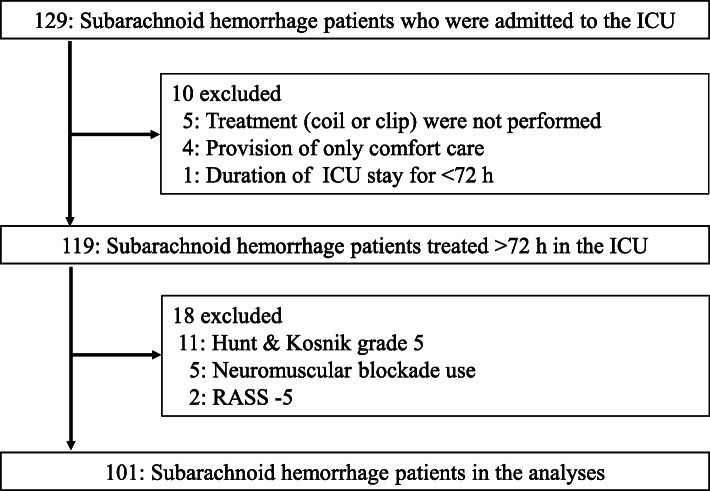
Patient flow

Among 129 patients with SAH who were admitted to the ICU, 119 met the inclusion criteria. Among those who met the inclusion criteria, 18 were excluded due to H & K grade 5 or RASS-5 without analgosedation or neuromuscular blockade use during the first 24–72 h after admission. The remaining 101 patients were ultimately included for analyses (Fig. 1).

### Baseline characteristics of the study population

The study population included 101 patients (median age, 65 years; 58 (57.4%) were women). Unfavorable neurological outcomes were observed in 52 patients (51.5%) (Table 1).

**Table 1:** Comparison of baseline characteristics according to physical restraint during the first 24 to 72 h after admission

Variables	Total (*n* = 101)	Physical restraint during the first 24 to 72 h after admission			*P* value
		No physical restraint (*n* = 46)	Intermittent physical restraint (*n* = 26)	Continuous physical restraint (*n* = 29)	
Age (year)	65 (48–77)	58 (42–73)	69 (51–74)	69 (62–81)	0.065
Age > 65 years (%)	50 (49.5)	18 (39.1)	16 (61.5)	16 (55.2)	0.145
Female sex (%)	58 (57.4)	30 (65.2)	17 (65.4)	11 (37.9)	0.042
Hunt & Kosnik grade	2.0 (2.0–3.0)	2.0 (1.0–3.0)	2.5 (2.0–3.0)	2.0 (2.0–4.0)	0.117
Fisher score	3 (3–3)	3 (2–3)	3 (3–3)	3 (3–3)	0.177
WFNS grade	2 (1–4)	2 (1–3)	2 (1–4)	2 (2–4)	0.117
Treatment modality (%)					
Endovascular	80 (79.2)	40 (87.0)	20 (76.9)	20 (68.9)	0.165
Surgical	21 (20.8)	6 (13.0)	6 (23.1)	9 (31.3)	
Laboratory data on admission					
Albumin (g/dL)	4.0 (3.6–4.3)	3.9 (3.5–4.5)	4.0 (3.7–4.3)	4.0 (3.8–4.3)	0.697
Glucose (mg/dL)	135 (115–167)	130 (112–157)	138 (122–190)	146 (113–173)	0.153
Lactate level (mg/dL)	11.0 (8.0–20.0)	10.0 (7.0–20.0)	11.0 (6.5–17.5)	13.0 (9.0–29.0)	0.208
Aneurysm location					
Anterior communicating/ cerebral artery aneurysm	32 (31.7)	14 (30.4)	9 (34.6)	9 (31.0)	0.165
Internal carotid artery aneurysm^a^	31 (30.7)	20 (43.5)	6 (23.1)	5 (17.2)	
Middle cerebral artery aneurysm	17 (16.9)	4 (8.7)	5 (19.2)	8 (27.6)	
Posterior circulation^b^ aneurysm	21 (20.8)	8 (17.4)	6 (23.1)	7 (24.1)	
Delayed cerebral ischemia (%)	21 (20.8)	10 (21.7)	3 (11.5)	8 (27.6)	0.335
Outcome at discharge (%)					
Favorable (mRS 0–2)	49 (48.5)	29 (63.0)	12 (46.2)	8 (27.6)	0.011
Unfavorable (mRS 3–6)	52 (51.5)	17 (37.0)	14 (53.9)	21 (72.4)	
Survive (%)	100 (99.0)	46 (100.0)	26 (100.0)	28 (96.6)	0.285
Duration of mechanical ventilator (day)	3 (2.0–11.0)	2 (1.0–3.0)	3 (1.8–6.0)	7 (3.5–14.0)	< 0.001
Length of ICU stay (day)	17 (15–19)	17 (13–19)	16 (14–17)	17 (15–19)	0.682
Length of hospital stay (day)	27 (23–37)	28 (22–36)	28 (24–35)	27 (23–42)	0.892
Duration of physical restraint (h)	11 (0–48)	0 (0–0)	20 (13–36)	48 (48–48)	< 0.001

Data are presented as medians (interquartile range, IQR) for continuous variables and *N* (percentage) for categorical variables

*WFNS* World Federation Neurological Surgeons, *mRS* modified Rankin scale, *ICU* Intensive care unit

^a^Internal carotid artery aneurysm: including posterior communicating region

^b^Posterior circulation: including the vertebral artery, basilar artery, cerebellar arteries, and posterior cerebral artery

Among them, 46 patients (45.5%) did not use physical restraint and 55 (54.5%) patients used physical restraint during the first 24–72 h after admission: 26 (25.7%) intermittent and 29 (28.7%) continuous. The median duration of physical restraint during the first 24–72 h after admission was 11 h (interquartile range, 0–48). Their distribution is shown in Supplemental Figure 1.

Baseline characteristics were compared according to three groups of physical restraint during the first 24–72 h after admission. Significant differences were observed in the outcome at discharge and duration of mechanical ventilator use. The proportion of unfavorable outcome at discharge was 37.0, 53.9, and 72.4% in patients with no, intermittent, and continuous use of physical restraint, respectively (Table 1). A comparison of baseline characteristics according to favorable and unfavorable outcomes is shown in Supplemental Table 1.

### Association between physical restraint and sedative and antipsychotic medication use

Among three groups, a significant difference was observed in incidences of propofol and DEX use (Table 2). Regarding the evaluation of RASS score, the differences in maximum and minimum RASS score, agitated state (RASS ≥ 1), and deep sedation (RASS score ≤ − 3) were also significant among the three groups. Differences in antipsychotic medications use were not significant (Table 2).

**Table 2:** Association between physical restraint and sedative and antipsychotic medications use

Variables	Total (*n* = 101)	Physical restraint during the first 24 to 72 h after admission			*P* value
		No physical restraint (*n* = 46)	Intermittent physical restraint (*n* = 26)	Continuous physical restraint (*n* = 29)	
Sedatives and analgesia (%)					
Midazolam	12 (11.9)	5 (10.9)	2 (7.7)	5 (17.2)	0.529
Propofol	30 (29.7)	4 (8.7)	10 (38.5)	16 (55.2)	< 0.001
Dexmedetomidine	33 (32.7)	5 (10.9)	10 (38.5)	18 (62.1)	< 0.001
Fentanyl	25 (24.8)	9 (19.6)	5 (19.2)	11 (37.9)	0.150
Evaluation of RASS					
Maximum RASS score	0 (0–1.0)	0 (0–0)	0 (− 0.3–1.0)	1 (− 1.0–1.5)	0.005
Minimum RASS score	− 2.0 (− 4.0 to − 1.0)	− 1.0 (− 1.0 to − 1.0)	− 2.5 (− 4.0 to − 1.0)	− 4 (− 4.0 to − 4.0)	< 0.001
RASS score ≥ 1 (%)	36 (35.6)	5 (10.9)	12 (46.2)	19 (65.5)	< 0.001
Duration of RASS score ≥ 1 (h)	0 (0–3.0)	0 (0–0)	0 (0–7.3)	2.0 (0–10.5)	< 0.001
RASS score ≤ − 3 (%)	49 (48.5)	9 (19.6)	13 (50.0)	27 (93.1)	< 0.001
Duration of RASS score ≤ − 3 (h)	0 (0–40.5)	0 (0–0)	0.5 (0–31.0)	36.0 (14.0–45.0)	< 0.001
Antipsychotic medications (%)	7 (6.9)	1 (2.2)	2 (7.7)	4 (13.8)	0.153
Number of devices^a^	7.0 (6.0–7.0)	7.0 (6.0–7.0)	7.0 (6.8–7.0)	7.0 (6.5–8.0)	0.436

*RASS* Richmond Agitation–Sedation scale

^a^Devices: endotracheal tube, central venous catheter, arterial line, peripheral venous catheter, nasogastric tube, urinary catheter, external ventricular drain, lumbar spinal drain, and intracranial pressure sensor

### Multivariable logistic regression analysis

Regarding the primary outcome, this study found that continuous use of physical restraint during the first 24–72 h after admission was significantly associated with unfavorable neurological outcomes at discharge in patients with SAH (odds ratio (OR), 3.54; 95% confidence interval (CI), 1.05–13.06; *p* = 0.042) compared with no physical restraint (Table 3).

**Table 3:** Unadjusted and adjusted associations between physical restraint and unfavorable (mRS3-6) outcomes

Variables	Univariate analysis		Multivariable analysis	
	OR (95% CI)	*p* value	OR (95% CI)	*p* value
Age (> 65 years)	5.62 (2.44–13.61)	< 0.001	8.62 (3.00–28.49)	< 0.001
Hunt & Kosnik grade^a^	2.18 (1.43–3.50)	< 0.001	2.48 (1.49–4.43)	< 0.001
Treatment modality (coil vs. clip)	1.55 (0.59–4.19)	0.374	1.03 (0.28–3.86)	0.962
Aneurysm location (ACA^b^ or not)	2.34 (0.995–5.737)	0.051	3.51 (1.12–12.47)	0.031
Physical restraint during the first 24 to 72 h after admission				
No physical restraint	Reference		Reference	
Intermittent physical restraint	1.99 (0.75–5.37)	0.165	1.05 (0.30–3.51)	0.943
Continuous physical restraint	4.48 (1.68–12.88)	0.002	3.54 (1.05–13.06)	0.042

*mRS* modified Rankin scale, *ACA* Anterior cerebral arteries, *OR* Odds ratio, *CI* Confidence interval

^a^Continuous variable

^b^Including the anterior cerebral artery and anterior communicating artery

### Association between physical restraint and delirium

**Fig. 2:**
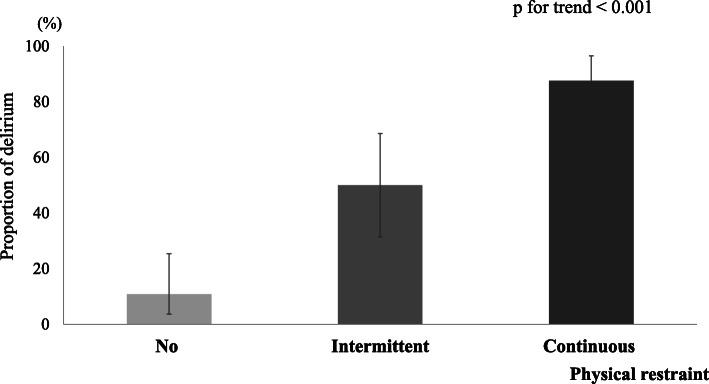
Associations between physical restraint during the first 24–72 h after admission and delirium during the first 48–72 h after admission. The proportion of delirium during the first 48–72 h after admission was 4 patients (10.8%) in the no physical restraint, 12 (50.0%) in the intermittent, and 21 (87.5%) in the continuous. Error bars indicate 95% confidence interval

Among all participants, delirium during the first 48–72 h after admission was assessed in 85 patients and was found in 37 patients (43.5%). A linear trend was observed in the development of delirium among three groups (4 patients (10.8%) in the no physical restraint, 12 (50.0%) in the intermittent, and 21 (87.5%) in the continuous; *p* < 0.001) (Fig. 2).

### Subgroup and sensitivity analysis

In patients with RASS of ≥ − 2 during the first 24 to 72h after admission, continuous use of physical restraint was significantly associated with unfavorable neurological outcomes at discharge than no physical restraint in patients with SAH (OR, 6.88; 95% CI, 1.69–32.36; *p* = 0.007). In patients with H & K grade (1–3) on admission, continuous use of physical restraint during the first 24 to 72h after admission was more significantly associated with unfavorable neurological outcomes at discharge than no physical restraint in patients with SAH (OR, 6.94; 95% CI, 1.66–33.92; *p* = 0.007) (Table 4). In the analysis using different definitions of physical restraint during the first 24–72 h after admission as a continuous variable, physical restraint was significantly associated with unfavorable outcomes (OR, 1.13; 95% CI, 1.01–1.28; *p* = 0.039; Table 4).

**Table 4:** Subgroup and sensitivity analysis of the association between physical restraint and unfavorable (mRS3-6) outcome

Models and variables	Univariate analysis		Multivariable analysis	
	OR (95% CI)	*p* value	OR (95% CI)	*p* value
**RASS ≥ − 2 (*n*** **= 82)**				
Physical restraint during the first 24 to 72 h after admission				
No physical restraint	Reference		Reference	
Intermittent physical restraint	3.02 (0.97–9.82)	0.057	1.58 (0.40–6.16)	0.509
Continuous physical restraint	8.29 (2.65–28.87)	< 0.001	6.88 (1.69–32.36)	0.007
**Hunt & Kosnik grades 1–3 (*n*** **= 81)**				
Physical restraint during the first 24 to 72 h after admission				
No physical restraint	Reference		Reference	
Intermittent physical restraint	2.64 (0.87–8.25)	0.088	1.92 (0.50–7.44)	0.338
Continuous physical restraint	7.25 (2.31–25.49)	< 0.001	6.94 (1.66–33.92)	0.007
**Different definition** (continuous variable)				
Physical restraint during the first 24 to 72 h after admission (OR per each 5 h)	1.18 (1.07–1.30)	0.001	1.13 (1.01–1.28)	0.039

Adjusted factors were the same as those during primary analysis: physical restraint requirement during the first 24 to 72 h after admission, age (> 65 years), Hunt & Kosnik grade, treatment modality (coil or clip), and aneurysm location (anterior cerebral arteries or not)

*mRS* modified Rankin scale, *OR* Odds ratio, *CI* Confidence interval

## Discussion

In this study, continuous use of physical restraint during the first 24–72 h after admission was found to be more significantly associated with unfavorable neurological outcomes at discharge than no physical restraint in patients with SAH. Continuous use of physical restraint during the first 24–72 h after admission was also associated with the occurrence of delirium during the first 48–72 h ICU stay.

A recent study performed by Reznik et al. demonstrated that a longer duration of agitation in the acute setting may be associated with more favorable outcomes in patients with SAH with RASS score > 0 [6]. This result seemed to be inconsistent with the results of the present study. Moreover, even in the subgroup analysis limited to patients with RASS ≥ − 2 during the first 24–72 h after admission in the present study, the stronger conclusion that continuous use of physical restraint during the first 24–72 h after admission was more significantly associated with unfavorable neurological outcomes than no physical restraint was obtained. Because Reznik et al. included only patients with RASS scores > 0 and assessed outcome at 3 months, the populations and outcomes of the two studies were not the same. In addition, because agitation was not evaluated directly in the present study, the discrepancy between two studies could not be confirmed precisely. Further study is required to establish whether the cause is due to a difference in analysis, a difference in race, or other factors.

The increasing exposure of patients to potentially harmful sedative and antipsychotic medications can be reduced with the use of physical restraint [14]; however, sedative drug use such as midazolam and propofol was paralleled to physical restraint in this study because the target sedation could not be reached even if the appropriate sedative dose was used. The use of sedatives decreased in parallel with the minimum RASS score, but increased in parallel with the maximum RASS score in this study. Other reasons include the fact that in the case of deepening sedation, physical restraint is also used because bedside nurses feel that patients should not be moved frequently. That feeling gets stronger, which is a psychological characteristic of Japanese. In neurocritically ill patients, delirium was independently associated with worse neurological outcome [7, 15], and continuous use of physical restraint was associated with the occurrence of delirium in the acute phase in this study. In the present study, information on delirium during the first 48–72 h after admission was obtained to clarify the phase differences between physical restraint requirement and development of delirium. Of the 37 patients with delirium during 48–72 h after admission, 31 (84%) had already required physical restraint, including intermittent and continuous restraints before 48 h after admission. Thus, continuous use of physical restraint during the first 24–72 h after admission was considered to be more associated with unfavorable neurological outcomes. Another hypothesis could be that the patients with no physical restraint had a shorter ventilation period than those in the two physical restraint groups. Thus, more patients with good consciousness levels after initial treatment might have been included in the no physical restraint group. However, in our subgroup analysis, the significant association between the physical restraint requirement and unfavorable outcome persisted in a limited number of patients with non-severe SAH (H&K grade 1–3).

Although physical restraint was the exposure, not the intervention in the present study, the hypothesis that continuous use of physical restraint during the first 24–72 h after admission was more significantly associated with unfavorable neurological outcomes than no physical restraint might be generated. In addition, our subgroup analysis results suggested that its association in patients with non-severe SAH (H&K grade 1–3) was stronger than primary analysis. However, application of physical restraint is closely associated with sedative and antipsychotic medication use [16–18]. Moreover, no RCT has explored the safety and efficacy of physical restraint use in critically ill adult patients [1]. Thus, further study to explore the safety and efficacy of physical restraint for patients with SAH will be required. In the study, establishing appropriate outcomes such as post-aSAH syndrome [19], and harmful sedative and antipsychotic medication use to prevent self-extubation, tube dislodgement, and/or medical device removal may be required.

This study has several limitations that should be addressed. First, this was a retrospective single-center study with a small sample size; hence, the possible selection bias and uncontrolled confounding factors should be considered. Moreover, some patients who received unrecorded physical restraints might have been categorized into the no physical restraint group, which could be a misclassification bias. Further RCT or prospective and multicenter studies are warranted to confirm our findings. Second, no physical restraint criteria have been established. Although the treatment management was decided by discussion at the conference, the person in charge of the neurointensive care unit was the same throughout the study period, and no major change was made in the treatment protocol for physical restraint and sedation. Third, the details of the devices for physical restraint were not examined because of the small sample size. Finally, as the primary end point was unfavorable neurological outcome at hospital discharge, differences in the time of evaluation may have affected the results. Further studies to evaluate with long-term outcomes are needed.

## Conclusions

This study found that continuous use of physical restraint during the first 24–72 h after admission was significantly more associated with unfavorable neurological outcomes than no physical restraint at discharge in patients with SAH.

## Supplementary Information

**Additional file 1: Supplemental Figure 1.** Distribution of physical restraint duration during the first 24–72 h after admission. Continuous physical restraint was defined as the patients who required continuous physical restraint during the first 24–72 h after admission. Intermittent physical restraint was defined as the patients who required any physical restraint during the first 24–72 h after admission.

**Additional file 2: Supplemental Table 1.** Comparison of baseline characteristics with favorable (mRS 0–3) and unfavorable (mRS 4–6) outcomes.

## Data Availability

The datasets used and/or analyzed during the present study are available from the corresponding author on reasonable request.

## Abbreviations

SAH: Subarachnoid hemorrhage
ICU: Intensive care unit
RASS: Richmond Agitation–Sedation Scale
DCI: Delayed cerebral ischemia
DEX: Dexmedetomidine
CAM-ICU: Confusion Assessment Method for the ICU
WFNS: World Federation Neurological Surgeons
mRS: Modified Rankin scale
OR: Odds ratio
CI: Confidence interval
