# Supplementary material for: Association between physical restraint requirement and unfavorable neurologic outcomes in subarachnoid hemorrhage
Source: J Intensive Care. 2021 Mar 12;9:24. doi: 10.1186/s40560-021-00541-z (PMC7952502; doi:10.1186/s40560-021-00541-z)
Supplement: Supplementary file 1 — Additional file 1: Supplemental Figure 1. Distribution of physical restraint duration during the first 24–72 h after admission. Continuous physical restraint was defined as the patients who required continuous physical restraint during the first 24–72 h after admission. Intermittent physical restraint was defined as the patients who required any physical restraint during the first 24–72 h after admission. [file 40560_2021_541_MOESM1_ESM.pptx]

## Slide 1
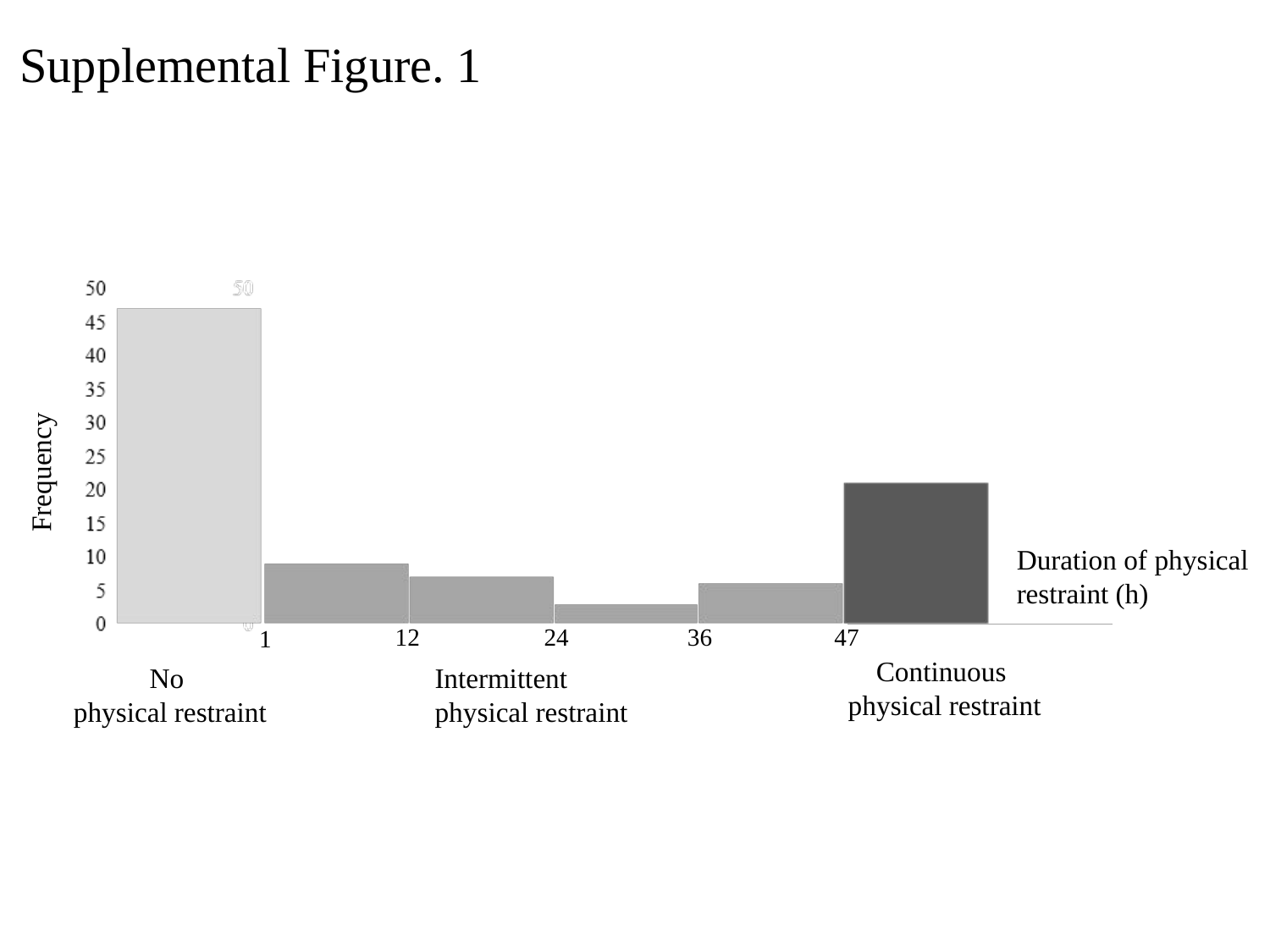

Supplemental Figure. 1
Frequency
Duration of physical restraint (h)
36
12
24
47
1
Continuous
physical restraint
No
physical restraint
Intermittent
physical restraint
