# Supplementary material for: Association between physical restraint requirement and unfavorable neurologic outcomes in subarachnoid hemorrhage
Source: J Intensive Care. 2021 Mar 12;9:24. doi: 10.1186/s40560-021-00541-z (PMC7952502; doi:10.1186/s40560-021-00541-z)
Supplement: Supplementary file 2 — Additional file 2: Supplemental Table 1. Comparison of baseline characteristics with favorable (mRS 0–3) and unfavorable (mRS 4–6) outcomes. [file 40560_2021_541_MOESM2_ESM.docx]

**Supplemental Table 1. Comparison of baseline characteristics with favorable (mRS 0**–**3) and unfavorable (mRS 4**–**6) outcomes**

|  | **Favorable mRS 0**–**2**  (n = 49) | **Unfavorable mRS 3**–**6**  (n = 52) | *P*-value |
| --- | --- | --- | --- |
| Age (year) | 54 (42–69) | 73 (63–81) | <0.001 |
| Age > 65 years (%) | 14 (28.6) | 36 (69.2) | <0.001 |
| Female sex (%) | 31 (63.3) | 27 (51.9) | 0.249 |
| Hunt & Kosnik grade | 2.0 (1.0–2.5) | 3.0 (2.0–4.0) | <0.001 |
| Fisher score | 3 (3–3) | 3 (3–3) | 0.225 |
| WFNS grade | 2 (1–2) | 2 (2–4) | 0.001 |
| Treatment modality (%) |  |  |  |
| Endovascular | 37 (75.5) | 43 (82.7) | 0.374 |
| Surgical | 12 (24.5) | 9 (17.3) |  |
| Laboratory data on admission |  |  |  |
| Albumin (g/dL) | 4.0 (3.7–4.5) | 3.9 (3.6–4.2) | 0.078 |
| Glucose (mg/dL) | 136 (115–158) | 130 (115–176) | 0.908 |
| Lactate level (mmol/L) | 10.0 (8.0–18.0) | 12.0 (7.3–21.5) | 0.430 |
| Aneurysm location |  |  |  |
| Anterior communicating/ cerebral artery aneurysm | 11 (22.4) | 21 (40.4) | 0.195 |
| Internal carotid artery aneurysm^b^ | 19 (38.8) | 12 (23.1) |  |
| Middle cerebral artery aneurysm | 9 (18.3) | 8 (15.4) |  |
| Posterior circulation^c^ aneurysm | 10 (20.4) | 11 (21.2) |  |
| Delayed cerebral ischemia (%) | 7 (14.3) | 14 (26.9) | 0.118 |
| Survive (%) | 49 (100) | 51 (98.1) | 0.329 |
| Duration of mechanical ventilator (day) | 2.0 (1.0–3.0) | 9.5 (3.0–16.8) | <0.001 |
| Length of ICU stay (day) | 15 (14–17) | 18(16–22) | <0.001 |
| Length of hospital stay (day) | 25 (22–30) | 33 (25–49) | <0.001 |
| Duration of physical restraint (hour) | 0 (0–20) | 33 (0–48) | 0.002 |
| No physical restraint (%) | 29 (59.2) | 17 (32.7) | 0.008 |
| Delirium (%) | 14 (29.1) | 23 (62.1) | 0.002 |
| Sedatives and analgesia (%) |  |  |  |
| Midazolam | 1 (2.04) | 11 (21.2) | 0.003 |
| Propofol | 9 (18.4) | 21 (40.4) | 0.016 |
| Dexmedetomidine | 8 (16.3) | 25 (48.1) | <0.001 |
| Fentanyl | 8 (16.3) | 17 (32.7) | 0.057 |
| Evaluation of RASS |  |  |  |
| Maximum RASS score | 0 (0–1) | 0 (−4–1) | 0.271 |
| Minimum RASS score | −1 (−2–−1) | −4 (−4–−1) | <0.001 |
| RASS score ≥1 (%) | 14 (28.6) | 22 (42.3) | 0.150 |
| Duration of RASS score ≥1 (hour) | 0 (0–2) | 0 (0–5) | 0.147 |
| RASS score ≤−3 (%) | 11 (22.5) | 38 (73.1) | <0.001 |
| Duration of RASS score ≤−3 (hour) | 0 (0–0) | 33.5 (0–48) | <0.001 |
| Antipsychotic medications (%) | 3 (6.1) | 4 (7.7) | 0.756 |
| Number of devices^a^ | 7 (7–8) | 7 (7–7) | 0.470 |

WFNS, World Federation Neurological Surgeons; mRS, modified Rankin scale; ICU, intensive care unit; RASS, Richmond Agitation–Sedation scale

Data are presented as medians (interquartile range, IQR) for continuous variables and *N* (percentage) for categorical variables.

^a^devices: endotracheal tube, central venous catheter, arterial line, peripheral venous catheter, nasogastric tube, urinary catheter, external ventricular drain, lumbar spinal drain, and intracranial pressure sensor.

^b^Internal carotid artery aneurysm: including posterior communicating region.

^c^Posterior circulation: including the vertebral artery, basilar artery, cerebellar arteries, and posterior cerebral artery.
